# Transcriptome changes in the developing sugarcane culm associated with high yield and early-season high sugar content

**DOI:** 10.1007/s00122-022-04058-3

**Published:** 2022-02-27

**Authors:** Virginie Perlo, Gabriel R. A. Margarido, Frederik C. Botha, Agnelo Furtado, Katrina Hodgson-Kratky, Fernando H. Correr, Robert J. Henry

**Affiliations:** 1 Queensland Alliance for Agriculture and Food Innovation, University of Queensland, Brisbane, QLD 4072 Australia; 2 Departamento de Genética, Escola Superior de Agricultura “Luiz de Queiroz”, Universidade de São Paulo, Piracicaba, São Paulo, 13418-900 Brazil; 3 The University of Queensland, Level 2, Queensland Bioscience Precinct [#80], 306 Carmody Road St Lucia, St Lucia, QLD 4072 Australia

## Abstract

Sugarcane, with its exceptional carbon dioxide assimilation, biomass and sugar yield, has a high potential for the production of bio-energy, bio-plastics and high-value products in the food and pharmaceutical industries. A crucial challenge for long-term economic viability and environmental sustainability is also to optimize the production of biomass composition and carbon sequestration. Sugarcane varieties such as KQ228 and Q253 are highly utilized in the industry. These varieties are characterized by a high early-season sugar content associated with high yield. In order to investigate these correlations, 1,440 internodes were collected and combined to generate a set of 120 samples in triplicate across 24 sugarcane cultivars at five different development stages. Weighted gene co-expression network analysis (WGCNA) was used and revealed for the first time two sets of co-expressed genes with a distinct and opposite correlation between fibre and sugar content. Gene identification and metabolism pathways analysis was used to define these two sets of genes. Correlation analysis identified a large number of interconnected metabolic pathways linked to sugar content and fibre content. Unsupervised hierarchical clustering of gene expression revealed a stronger level of segregation associated with the genotypes than the stage of development, suggesting a dominant genetic influence on biomass composition and facilitating breeding selection. Characterization of these two groups of co-expressed key genes can help to improve breeding program for high fibre, high sugar species or plant synthetic biology.

**Supplementary Information:**

The online version contains supplementary material available at 10.1007/s00122-022-04058-3.

## Introduction

Sugarcane is a tropical and subtropical perennial grass typically harvested once a year in commercial applications, but cultivar management allows harvesting at different times of the year with significant impacts on industry profitability (Di Bella et al. 2008).

This C4 plant has the most significant crop production quantity, reaching 1.9 billion tonnes in 2018 (FAOSTAT 2020), and the highest maximum efficiency of converting solar energy to biomass (Henry 2010). This capability to accumulate very high levels of sucrose in the culm (Lingle and Thomson 2012) associated with exceptional biomass yield, mean sugarcane is well-designed as a crop for a renewable energy alternative to fossil fuels (Alexander 1985; Tilman et al. 2006; Renouf et al. 2008; Waclawovsky et al. 2010).

The sugarcane genome is interspecific hybrids highly polyploids and aneuploids (Piperidis and D’Hont 2020). Despite this challenging complexity, significant research and breeding have been undertaken to produce new sugarcane varieties to improve productivity (Botha 2009; Yadav et al. 2020). Improving breeding using molecular markers to optimize trait selection is an important focus to increase early-season sugar content and fibre content to extend crop harvesting. The sugar dynamics between source and sink perhaps contribute to growth and sucrose accumulation (Roopendra et al. 2019).

Many genes are involved in sucrose synthesis and accumulation. Sucrose synthase (SuSy), sucrose-phosphate synthase (SPS), soluble acid (SAI) and cell wall invertases (CWI) have been intensely studied (Chandra et al. 2015; Li et al. 2019; Thirugnanasambandam et al. 2019; Botha and Black 2000; Singh et al. 2021). Relation between sucrose and hexoses level with the activities of invertases sucrose synthase and sucrose-phosphate synthase at different development stages have been described (Batta et al. 2008). Neutral Invertase (NI, EC 3.2.1.26) has been reported to play a major role in sugar accumulation and to be more abundant in mature internodes (Rose and Botha 2000; Rossouw et al. 2010). Manipulation of invertase activity was reported to affect sucrose metabolism and response to biotic and abiotic stresses (Shivalingamurthy et al. 2018).

But currently, the identification of genes specifically linked to early-season sugar content has not been reported. Associating gene expression and metabolic pathways may give answers to clarify the interconnected regulatory networks associated with cell wall and sugar accumulation. This analysis could lead to enhance the difficult approach to produce cultivars targeting high sucrose and high fibre content (Jackson 2005). Fibre and sugar content are mainly stage dependant, with changes in carbon allocation, into protein and fibre during the early stage in immature tissue and to sucrose storage in mature culm (Bindon and Botha 2002; Botha and Mcdonald 2010; Lingle and Smith 1991; Lingle and Thomson 2012; Van Der Merwe et al. 2010; Van Der Merwe and Botha 2013; Wang et al. 2013; Whittaker and Botha 1997).

Harvest management has been enhanced by the selection of varieties such as KQ228 and Q253, utilized in the industry to allow harvesting and processing to be conducted over a longer period. These varieties are characterized by a high early-season sugar content associated with high final yield measured as tonnes of cane per hectare (Plunkett, 2013; Thirugnanasambandam et al. 2019; Perlo et al. 2020). Providing reliable specific biomarkers to identify these characteristics at the earliest possible growth stage will assist breeders to respond to this industry need. Transcriptomic markers could provide a blueprint for a better understanding of carbon partitioning to control sugar and fibre accumulation. Considering the high level of genome complexity, genomic analysis in support of breeding is particularly challenging in sugarcane (Ferreira et al. 2016; Hoang et al. 2017a). Transcriptome analysis has been widely used and proven to be an informative and highly reliable approach (Marioni et al. 2008). Analysing the transcriptome with RNA sequencing (RNA-seq) using next-generation sequencing (NGS) is a powerful technology for a plant with an incomplete reference genome (Conesa et al. 2016). Sugarcane reference genome is yet to be completed, and the sorghum genome is often used as reference genome (Thirugnanasambandam et al. 2018; Xu et al. 2018). Recently, the first mosaic monoploid reference of the sugarcane genome with 382 Mb has been released (Garsmeur et al. 2018). Co-expression networks analysis is an approach frequently used to explore the relationships between genes and phenotypic traits. Weighted gene co-expression network analysis (Langfelder et Horvath 2008) is widely used in medical science, for example, in breast and colon cancer research (Le et al. 2019; Pournoor et al. 2020) and in plants in diverse species such as barley, Arabidopsis, bamboo and rice (Childs et al. 2011; Hongjun et al. 2018; Liu et al. 2019). In this study, differential gene expression and co-expression networks were investigated across five developmental stages in 24 sugarcane cultivars to identify genes and metabolic pathways associated with high early sugar content and fibre content.

## Materials and methods

### Plant materials, field design and phenotypic measures

A selection of 24 sugarcane cultivars (KQ236, KQB09-20,432, MQ239, Q124, Q135, Q138, Q151, Q155, Q157, Q186, Q200, Q208, Q237, Q238, Q240, Q241, Q253, QN05-1743, SRA1, SRA2, SRA3, SRA5, SRA8) varying in sugar accumulation rates and fibre content with three replicates were planted in August 2017 at the Sugar Research Burdekin Station in Burdekin, QLD (19°34′08.0″S 147°19′30.7″E) and harvested in September 2018. Standard industry recommendations fertilization (N 160 kg/ha, P kg/ha, K kg/ha and S 20 kg/ha) with a 7-day flood irrigation was used. Three replicates per genotype were subject to identical environmental growing conditions. Cultivars were planted in 6 × 4 Latin square design with 4 m of cane with a 1.52-m row spacing. Samples were analysed with a modified method (Berding 2010). After clarification, Brix and polarity were measured at three different time points, in May, June and September 2018 (Perlo et al. 2020) and commercial cane sugar (CCS) was calculated. Detailed of sugar content, fibre content and genetic entities for these 24 cultivars (Table S1) are described in Perlo et al. (2020).

### Sample collection and processing for transcriptomic analysis

The samples were collected after 19 weeks and after 37 weeks. From four stalks of each genotype, three replicates of internodes 5 and 8 were collected, the 3rd and 6th internodes below the first visible dewlap leaf, respectively. Internodes 5, 8 and “Ex5” were collected during the second collection. During the first collection, internodes 5 were tagged on stools to be collected during the second collection as internode “Ex5” which was the internode in the mature plant. After excision samples were frozen in liquid nitrogen and pulverized as described in Perlo et al. (2020).

### RNA extraction

Total RNA was extracted from approximately 2.5 g of frozen powder for the three biological replicates of each genotype, using TRIzol reagent (Invitrogen). The supernatant containing the RNA was treated with Qiagen RNeasy Plant Minikit (Qiagen) for RNA purification (Furtado 2013; Henry and Furtado 2014). The RNA concentration was measured by spectroscopy with NanoDrop (Thermo Scientific).

RNA integrity was assessed with Agilent Bioanalyser. A260/280 ratios were measured, their values were around 2.1 and not below 1.8, making these samples accepted as “pure” RNA. RNA concentration and quality were checked on gel, using 0.7% agarose gel electrophoresed at 100 V for 60 min. Total extracted RNA samples were labelled and stored in a -80 °C freezer. For RNA-seq, the total RNA from the four stools of each tissue (internode 5, internode 8 and internode “ex-5”) of each genotype of each replicate were pooled in equimolar concentration, and 3 µg of RNA was used for library preparation and sequencing.

### RNA library for Illumina sequencing

The cDNA Illumina sequencing library preparation was processed using TruSeq RNA Library Preparation Kit with Ribo-Zero Plant (Illumina).

### Differential gene expression analysis

Using CLC Genomics Workbench 12.0.3, adaptors and low-quality bases were trimmed from raw reads with a cut-off of 0.01 and quality control on raw sequence data were assessed. The FASTQ sequence reads were aligned to sugarcane transcriptome references generated using long read (PacBio) sequencing (Hoang et al. 2017a). The following step was to generate a matrix of counts, transcripts per kilobase million (TPM) of 107,598 genes for the 360 samples.

The count table (TPM) was loaded in OmicsBox 1.1.164 where the differential gene expression (DGE) analysis was generated with a false discovery rate (FDR) p-value ≤ 0.05 and log fold change (FC) abs value > 1, trimmed mean of M values (TMM) normalization method, fitted the model using generalized linear models (GLM), statistical test and performed likelihood ratio (LR) test. DGE between different development stages were systematically compared (FDR-adjusted *p* value ≤ 0.05).

## Characterization of the genotype x internode interaction

### RNA-seq data pre-processing, de novo assembly and quantification of expression levels

The sugarcane transcriptome reference based on PacBio long reads by Hoang et al. (2017a, b) provides a wide catalogue of (full-length) transcript isoforms and is a valuable resource for gene expression studies. RNA-seq with high-depth Illumina short reads can supplement this reference due to its broader sampling of the transcripts present in the libraries – 24 genotypes and five developmental stages. Thus, de novo transcriptome was assembled for the 360 sugarcane samples and used it as a reference to characterize the main effects of genotypes and internodes on gene expression profiles, as well as the interaction between these two factors. Trimmomatic v0.39 (Bolger et al. 2014) was used to remove Illumina adapters, the leading 13 bp of each read, bases with quality score less than 30 and reads shorter than 70 bp after trimming. Next, BBTools v38.79 (Bushnell 2014) was applied to remove contaminant ribosomal RNA reads, using the SILVA database (Quast et al*.*, 2013) and a *k*-mer size of 31 bp. Transcriptome de novo was assembled with Trinity v2.10.0 (Grabherr et al. 2011), in stranded mode and with normalization by read set, discarding contigs shorter than 300 bp. The final assembled transcripts were annotated with Trinotate v3.2.0 (Bryant et al. 2017) to assign the most likely hits from the UniProt database (UniProt, 2019).

Salmon v1.2.0 (Patro et al. 2017) was used to measure the abundance of each transcript in the de novo assembled transcriptome, by applying the *quasi-mapping* strategy on the high-quality RNAseq reads with GC bias correction turned on. Next, R package tximport (Soneson et al. 2016) was used to collect transcript-level abundances and obtain estimates of expression levels for each putative gene. Finally, RNAseq libraries were normalized and the genes filtered with the edgeR package (Robinson et al. 2010). To remove lowly expressed genes, only those with a count of 10 or more reads for at least 70% of the 360 samples were retained. Final estimates of gene expression were exported for use in the subsequent analysis as ${\text{log}}_{2}\left(CPM+1\right)$, where $CPM$ represents the normalized counts per million reads of each gene in each sample.

### Three-way analysis of gene expression

Tucker3 model (Tucker 1966), also known as three-way principal component analysis (PCA), was used to decompose the total variation in gene expression into components associated with genotypes, genes, and internodes. The average normalized expression levels are arranged in a three-dimensional array ${\text{X}}$ of dimensions $I\times J\times K$, such that the first dimension represents the $I$ samples (in this case the genotypes), the second dimension corresponds to the $J$ variables (genes) and the third dimension represents the $K$ conditions (internodes). Each element ${x}_{ijk}$ thus represents the expression level of the $j$ th gene, for the $i$ th genotype and internode $k$. The Tucker3 model decomposes the expression levels in three modes as follows:

$${x}_{ijk}={\sum }_{p=1}^{P}{\sum }_{q=1}^{Q}{\sum }_{r=1}^{R}{a}_{ip}{b}_{jq}{c}_{kr}{g}_{pqr}+{e}_{ijk}$$

where $i=1,\dots ,I$, $j=1,\dots ,J$ and $k=1,\dots ,K$. Coefficient ${a}_{ip}$ is the loading (score) of the $i$ th entity on the $p$ th component of the first mode. These ${a}_{ip}$ elements can be arranged in a component matrix ${\text{A}}$ corresponding to genotypes, of dimensions $I\times P$, where $P<I$, such that the $P$ components compress the information for this mode. Similarly, coefficients ${b}_{jq}$ and ${c}_{kr}$ represent loadings associated with genes and internodes. They can be arranged in component matrices ${\text{B}}$ and ${\text{C}}$, of dimensions $J\times Q$ and $K\times R$, respectively, with $Q<J$ and $R<K$. The coefficient ${g}_{pqr}$ is called the core component and measures the strength of association between components $p$, $q$ and $r$ of the three modes. In other words, it weighs the interaction among the corresponding components. Finally, ${e}_{ijk}$ indicates the error term. For a more detailed explanation of the Tucker3 model, including a visual representation of the component matrices, we refer the reader to Conesa et al. (2010).

To fit this model, the gene expression estimates were centred but not scaled, such that more highly expressed genes had a larger weight on the model. First the R package CA3variants (Lombardo et al. 2020) was used to choose the optimal number of components in each mode. The maximum number of components tested were: five components for the genotype mode, 20 components for the gene mode and three components for internodes. All 300 combinations were fitted, and models were assessed by the total explained variation of gene expression levels. The best model was chosen through visual inspection of the scree plot. Based on this evaluation, we fitted the final model with R package ThreeWay (Giordani et al. 2014) and provided plotting functions.

## Weighted gene co-expression network analysis (WGCNA)

### Data pre-processing

The transcripts per kilobase million (TPM) matrix of counts generated by CLC Genomics Workbench 12.0.3 profiled the expression of the transcripts of the 360 samples. The dataset contained 24 genotypes across five different developmental stages. We removed genes with more than 80 percentage of zero counts in all samples. Genes without any annotation from Blast2Go were filtered out. The expression profiling was determined for 77,755 annotated genes. TPM values were transformed into Log2 (TPM + 1).

### Weighted correlation network analysis (WGCNA)

WGCNA, R package version 1.69 (Zhang et Horvath, 2005; Langfelder et Horvath, 2008) was performed as previously described (Perlo et al. 2020). Considering the scale-free topology characteristic of the network (R2 = 0.82), the soft thresholding power β = 4 was selected. Minimum module size to be used in the module detection procedure selected was 500, with unsigned network type. All hierarchical clustering tree (dendrogram) were generated using ward.D linkage method.

### Gene identification

A combination of multiple annotation methods was used to cover larger functional and metabolic networks. Gene Ontology (GO) functional classification, using WEGO (Ye et al. 2018), was processed to compare the co-expressed genes of selected eigengene modules (ME) generated with WGCNA.

Functional annotation based on orthology (similar ancestral sequence across species) assignments or cluster of orthologous groups (COG) was performed with Eggnog-mapper v1 (Huerta-Cepas et al. 2016, 2019).

KEGG pathway enrichment analysis was processed using KEGG Orthology-Based Annotation System (KOBAS) on the genes of modules of interest (Xie et al. 2011).

Genes assigned to each of the eigengene modules from the same colour were co-expressed or connected, correlated with their gene expression profiles. The eigengene significance measured the connectivity of this group of genes to a trait of interest. The module significance to a trait was the average of absolute gene significance measure for all the genes of this module. This means that individual gene may be positively or negatively correlated to the traits.

Identification of genes specifically correlated to high sugar content and to high fibre content was realized using most significant co-expressed genes with the highest correlation coefficient (eigengene significance). Genes from the module (black) associated with early, mid-season sugar content and not assigned to the module (pink) correlated to fibre content were pre-selected. One hundred genes with the highest gene significance (independently of their modules) to early, mid-sugar content generated with WGCNA, were identified (Table S2). From this list, the preselected genes associated with the black module (sugar content) and not to pink module (fibre content) were matched and selected. This restrictive list represented specific co-expressed genes linked to sugar content (Table S3). Similar filtration was realized to generate a list of highly specific co-expressed genes linked to fibre content (Table S4 and Table S5). These specific genes with highest significance were annotated using OmicsBox 1.3. The workflow of data-processing, weighted correlation network analysis and gene identification is illustrated in Figure S1.

## Results

### Differential expression analysis during different developmental stages

**Fig. 1:**
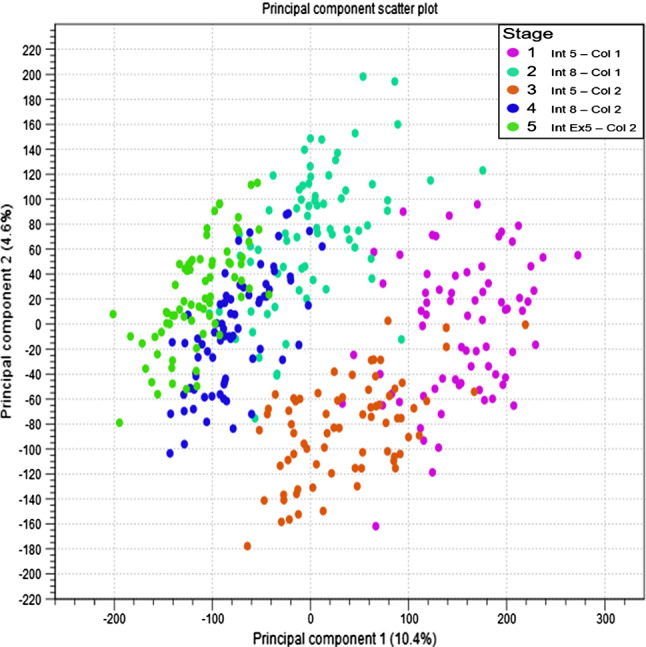
PCA analysis for RNA-seq for the different development stages (CLC Genomics Workbench 12.0.3). The PCA showed a distinct repartition of the samples according to the five development stages. In fuchsia (1), internode 5 from the first collection, in red (3) internode 5 from the second collection. In jade green (2), internode 8 from the first collection, in blue (4) internode 8 from the second collection, in bright green (5) internode Ex-5 from the second collection

Principal component analysis (PCA) was used to assess the correlation between the samples. This analysis revealed five distinct clusters, representing the five development stages (Collection 1, internode 5 and 8; and collection 2, internode 5, 8 and Ex-5) (Fig. 1). PCA1 which explained 10.4% of the overall variance between the development stages. The largest variation in PCA1 is between Int5 (youngest internode) and Int Ex5 (oldest internode). PCA2 explained 4.6% of the total variance and best separated Int 8 (Col1) and Int5 (Col2).

The internode 5 samples at two different collection points were separated by both PCA1 and PCA2 (Fig. 1). This illustrates that crop age and environmental factors also influence gene expression patterns.

**Fig. 2:**
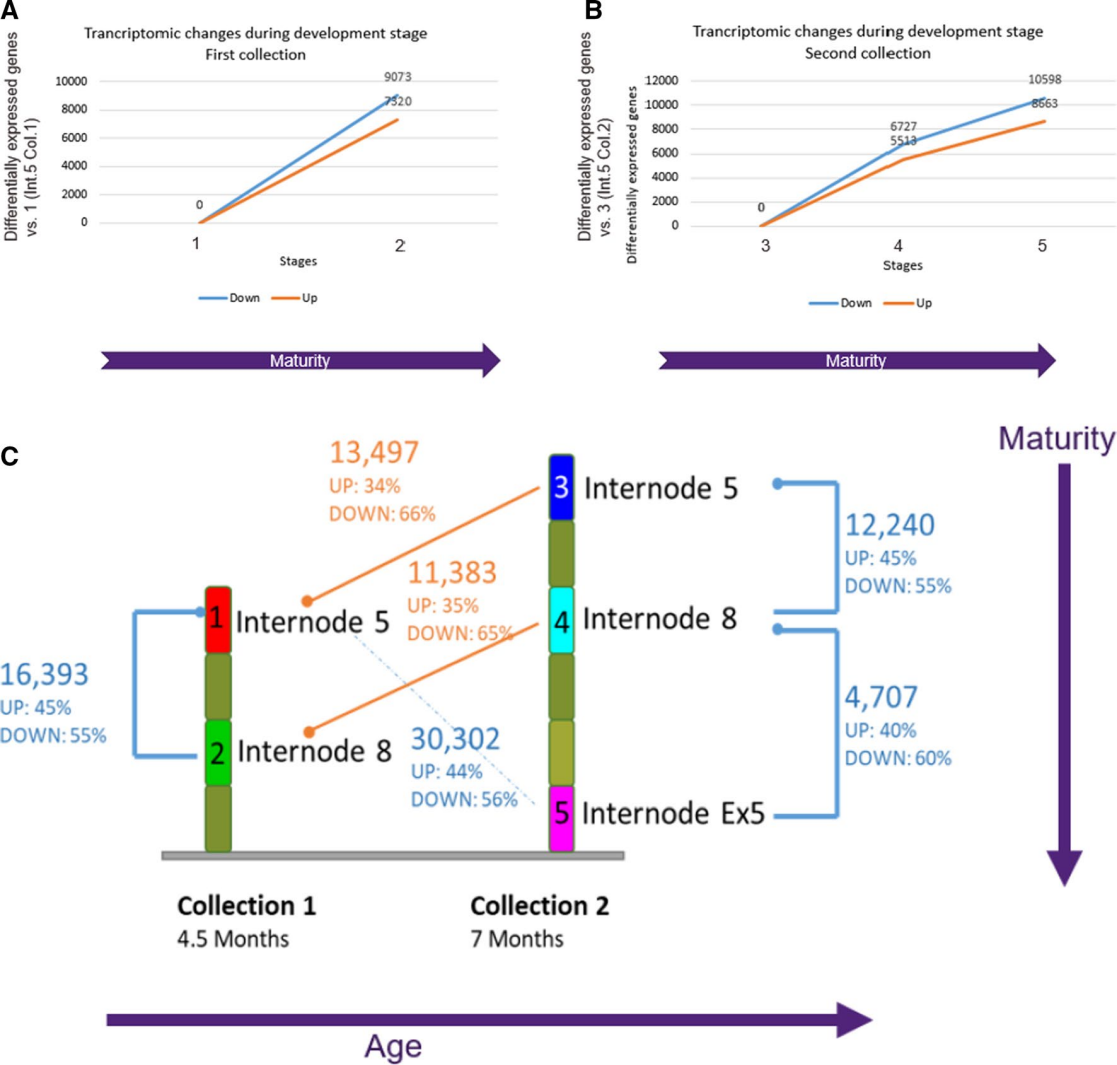
Differentially expressed genes between different development stages, with stage 1: internode 5, first collection, stage 2: internode 5, first collection, stage 3: internode 5, second collection, stage 4: internode 8, second collection and stage 5: internode Ex-5, second collection. **A** First collection (19 weeks)**, B** second collection (37 weeks). **C** Differentially expressed genes comparison between different stages of development. Reference conditions are displayed with a dot

Many genes were identified with significant variation in expression levels when comparing the development stages. For example, the number of differentially expressed genes (DEG) was highest when comparing between young internodes, such as internode 5 and 8 in the first period (19 weeks). In this case, 16,393 genes were differentially expressed. More genes, 9,073 genes were down-regulated while 7,320 genes were up-regulated (Fig. 2A). In contrast, during the late period (37 weeks) the number of DEG between mature internode 8 and Ex-5 were 3.5 lowest compared to the DEG between youngest internodes. In this case, only 4,707 genes were differentially expressed with 60% of genes down-regulated (Fig. 2B). Comparing the oldest (37 weeks) bottom mature internode Ex-5 with the young (19 weeks) top immature internode 5 revealed 19,261 genes differentially expressed with 10,598 down-regulated genes, and 8,663 up-regulated genes (Fig. 2C).

### Transcriptome analysis across genotypes and development

**Fig. 3:**
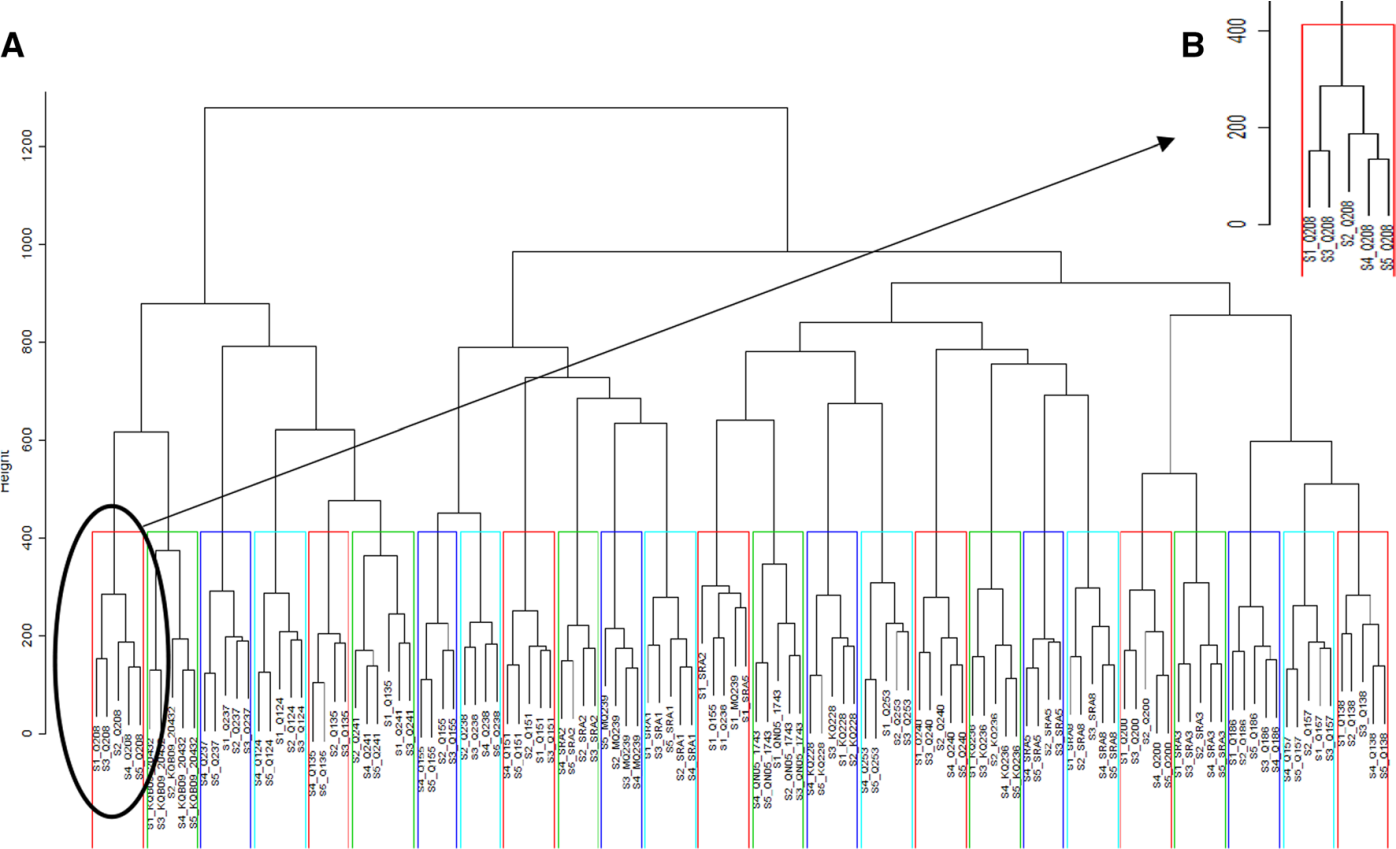
**A** Hierarchical clustering of gene expression for 24 genotypes at 5 different stages (S1, S2, S3, S4 and S5). Clusters were based on -log10 (means TPM + 1). Three replicates (*n* = 3) were used to calculate the mean for each genotype per stages. **B** Detail of the first cluster of the dendrogram of genome expression, illustrating a second level of hierarchy, per development stages

Hierarchical clustering of gene expression was generated with WGCNA. The dendrogram revealed two levels of segregation. The first level displayed a clear separation between the 24 genotypes, with each of the samples distinctly assigned to their own cultivars. For each of these groups, another level of hierarchy was identifiable, following a pattern with the separation by development stage, with two clusters regrouping the stages 1 and 3 and the stages 2, 4 and 5 (Fig. 3B). This separation reflected the results of the PCA in Fig. 1 with the clear separation of the five development stages and with more similarity between internode 5 (at stage 1 and 3) and between internode 8 (at stage 2 and 4).

Two main clusters were displayed (Fig. 3A). The first cluster (on the left) exhibited two sub-groups, the first one represented by Q208 and KQB09-20,432 and the second one included the old hybrid Q124 and Q135 from the same two parents NCo310 and QN54‐7096 (Cox 1995; Hogarth and Berding 2005; Hodgson-Kratky, 2020) with Q237 and Q241. In the second principal cluster (on the right), three sub-groups were separated, KQ228 is in the ‘middle’ cluster of this second group, including three other high early-season varieties, Q253, Q240 and SRA8. This cluster also included SRA2, SRA5, KQ236 and QN05-1743. These results revealed a strong similarity of gene expression between early-season cultivars KQ228, Q253, Q240 and SRA8. In the third sub-group, hybrids produced in 1970–1980, such as Q138, Q157 from the same two parents QN58-829 and QN66-2008 and Q157, Q186 and Q200 with the same male parent QN66-2008 were grouped together.

### Characterization of the genotype x internode interaction

After pre-processing and filtering of lower quality, RNA-seq reads de novo assembly was processed. The final transcriptome contained a total of 755,009 transcripts. After filtering out lowly expressed genes, which include assembly artefacts stemming from the large complexity of the sugarcane libraries, the final subset contained 38,365 transcripts, of which 19,903 were functionally annotated.

**Fig. 4:**
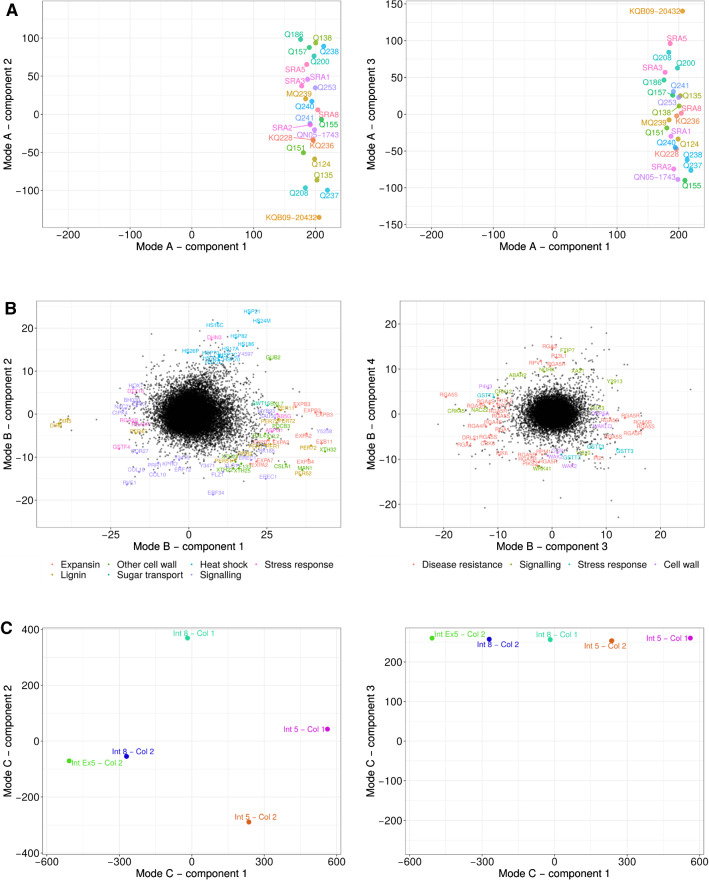
Loadings for the three modes of the Tucker3 model. Panel **(A)** at the top shows loadings for the genotype mode; panel **(B)** shows loadings for the gene mode, with some of the most extremely ranked genes annotated with different colours; and panel **(C)** at the bottom shows loadings for internodes. Note that the panels show different component combinations for each mode

The best Tucker3 model chosen included five, six and three components for the genotype, gene and internode modes, respectively. This final model explained 49.1% of the total variation in gene expression levels. Of the variance accounted for by the model, 55.1% was due to core element ${g}_{111}$, that is, the combination of the first component of mode A (herein denoted by A1), component B1 and component C1. The core component with the next largest value was ${g}_{122}$, which again included component A1 and accounted for 17.56% of the variance explained by the model. Inspection of the loadings in component A1 showed similar scores for the 24 genotypes, indicating that all genotypes had similar expression profiles for the genes associated with these core elements (Fig. 4A).

In that case, gene loadings in component B1 were associated with component C1, while loadings in B2 related to component C2, regardless of the genotype. Interestingly, components C1 and C2 closely matched the pattern seen in the PCA, with C1 revealing a gradation from immature (positives values) to mature internodes (negative values), and component C2 separated internode 5 in the second collection from internode 8 in the first collection (Fig. 4C). Genes with positive values in B1 were thus more highly expressed in immature internodes, and the most extreme values included many genes important for cell wall biosynthesis and modification, such as expansions, peroxidases that are involved in lignin metabolism xyloglucan endotransglucosylase-hydrolases and other transferases and hydrolases. These genes are identified with prefixes EX, PER and XTH in the left panel of Fig. 4B. B1 was also enriched with signalling-related genes such as kinases and transcription factors. In most cases, these were associated with negative loadings. The transcription factor NAC71, which is involved in abiotic stress responses, a glutathione S-transferase and a couple of resistance gene analogues, or RGAs are highlighted (Fig. 4B). Genes with higher expression in more mature internodes also included one peroxidase and two dirigent proteins, which are also related to lignin biosynthesis (Davin and Lewis 2000).

Transcripts with large positive values in B2 such as heat shock proteins (Hs genes) were more abundant in internode 8 (Col1) than in internode 5 (Col2), second collection (Fig. 4B, left panel).

While the previous two components were associated with variation among internodes, the next core elements ${g}_{233}$ and ${g}_{343}$ were responsible for 8.09% and 6.15% of the variation explained in the Tucker3 model, respectively. Both the ${g}_{233}$ and ${g}_{343}$ elements include the component C3, for which the five internodes showed similar loadings (as seen by the horizontal alignment of internodes on the right panel of Fig. 4C). Hence, these two elements correspond to variation in gene expression among genotypes, with similar profiles for all internodes. Loadings in component A2 (Fig. 4A, left panel) showed a pattern of separation among genotypes that agreed with the hierarchical clustering in Fig. 3, such that genotypes with negative loadings in A2 correspond to those on the left side of the dendrogram. On the other hand, component A3 separated genotypes in a way that those with large positive values included the fibre-rich ones, such as KQB09-20,432, SRA5 and SRA3, while those with higher sugar levels were in the opposite end, such as Q155 and KQ228 (right panel of Fig. 4A).

Component B3 of the gene mode was associated with component A2, while component B4 was associated with A3. In both cases, most genes with extreme loadings were annotated as RGAs and other disease resistance proteins, such as Pik and R proteins—RPV, RPM and RPP (Fig. 4B, right panel). We also observed genes coding for glutathione S-transferase and different signalling molecules, such as transcription factors WRK41 and NAC22, and other genes involved in stress response and hormone signalling, in particular abscisic and jasmonic acids. These included a 14–3-3-like protein, cysteine-rich receptor-like protein kinase 45, abscisic acid hydrolase and an FT-interacting protein. Given the importance of selection for disease resistance in sugarcane breeding programs, it is not surprising to identify this large number of differentially expressed genes among this set of selected sugarcane genotypes.

The most extreme genes in component B3 also included cell wall-related genes, such as wall-associated kinases, microtubule-associated protein 5A and cinnamoyl-CoA reductase, which is important for lignin synthesis. Among the genes identified in Fig. 4B, we highlight the phytochrome interacting factor-like (PIL13).

### Weighted gene co-expression network analysis

**Fig. 5:**
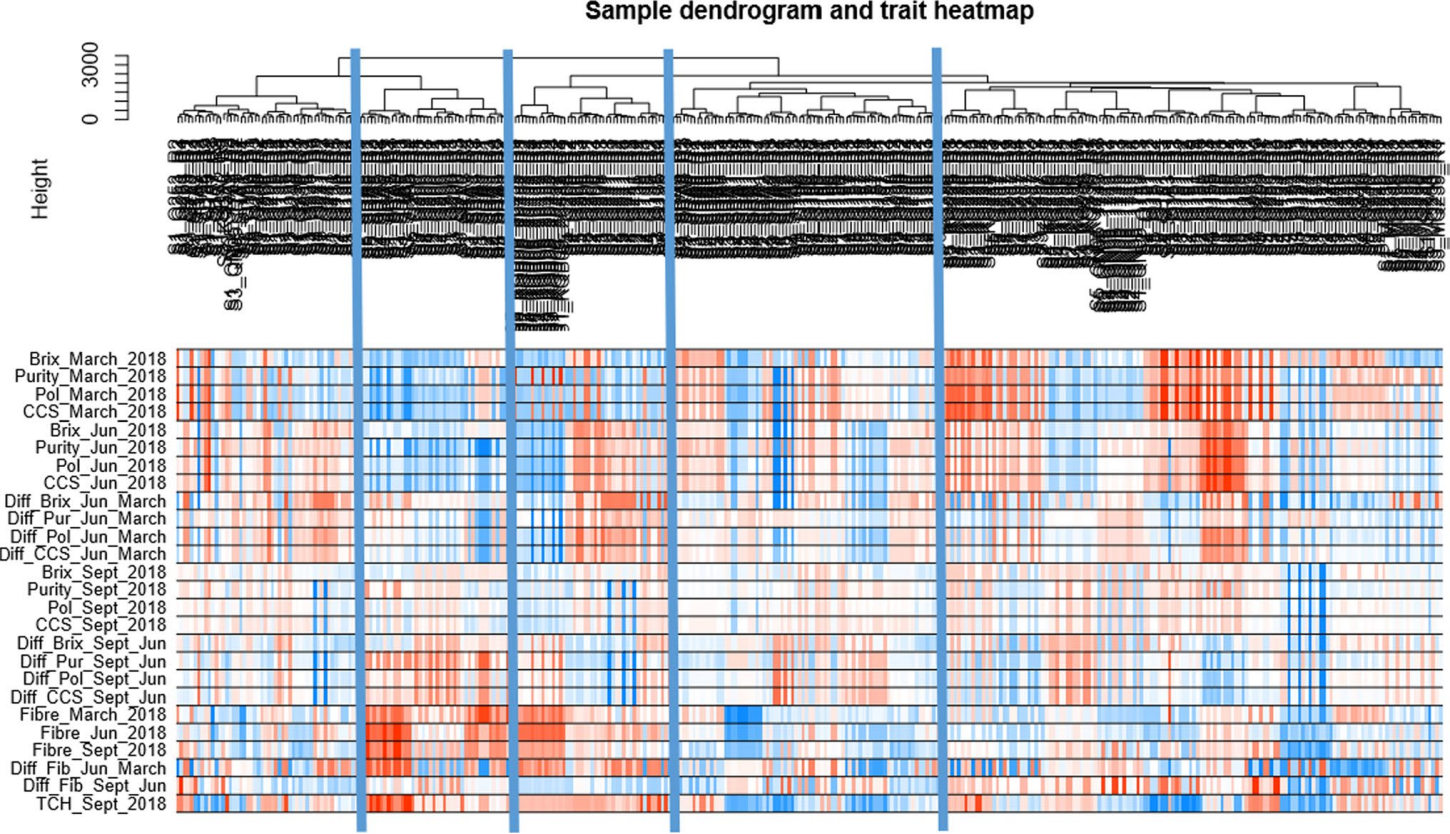
Sample dendrogram and trait heatmap generated with WGCNA. The clustering on the top was based on the -log(TPM + 1) values per genes for the 360 samples. The heatmap, with colour intensity, was proportional to Brix, purity, polarity, CCS, fibre levels and respective difference for early, mid- and late season and tons of sugarcane per hectare (TCH). Traits data were normalized using Auto Scaling method. The red and blue colours represented, respectively, positive and negative. Clustering dendrogram of samples was based on Euclidean distance

A weighted gene co-expression network was constructed from 77,755 genes (Fig. 5), the hierarchical cluster tree of the 360 samples was associated with the traits heatmap. The sample dendrogram revealed an association between gene expression and traits.

Co-expression modules or Module Eigengene (ME) represented regrouped co-expressed genes assigned to the same colour. The grey module-combined genes not assigned to any co-expressed gene modules.

This module trait relationship analysis revealed ten modules. Four of these modules, the pink, black, red, and grey modules, were significantly correlated—positively or negatively—to early-season sugar content and inversely correlated to fibre content. The pink and red module showed a high positive correlation to fibre content and TCH with a significative negative correlation with mid-season sugar content and less so for early-season sugar content.

This module trait relationship displayed a high correlation between the black module and early, mid-season sugar content with a negative correlation with fibre and TCH.

**Fig. 6:**
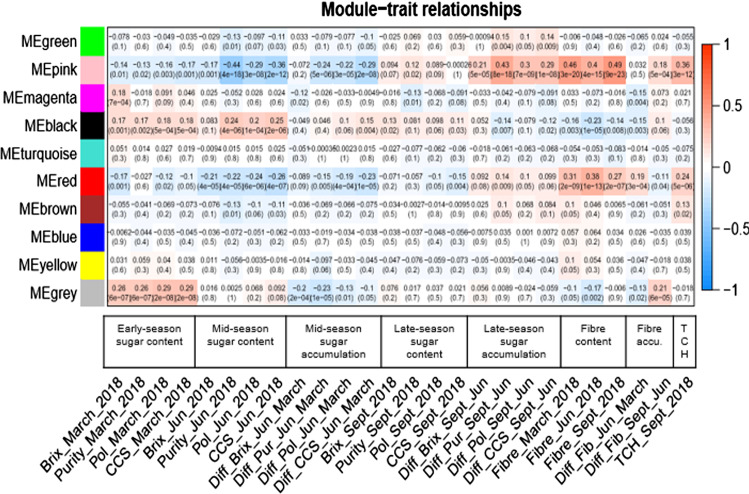
Module trait relationship. Heatmap of the correlation between essential agro economic traits as Brix, CCS, purity, polarity and TCH at different seasons and module eigengenes (ME). Positive and negative correlation were, respectively, represented in red and blue. Each cell contains the module trait correlation and in brackets p-value corresponding

The grey module of 15,929 genes not co-expressed was significantly linked to early sugar content and negatively correlated to fibre content (Fig. 6).

## Gene identification

### KEGG enrichment analysis

Enrichment pathways were generated for each of the modules (ME). This enrichment analysis revealed the cleavage of the modules of co-expressed genes based on their KEGG pathways. The grey module was linked to early-season high sugar content and identified multiple co-expressed pathways such as fatty acid biosynthesis, starch and sucrose, cyanoamino acid, galactose metabolism and pantothenate and CoA biosynthesis. The black module was linked to high early-mid-season sugar content and relied on co-expressed pathways such as ABC transporters, RNA transport, 2-oxocarboxylic acid metabolism, mRNA surveillance and citrate cycle TCA. The pink module linked to high fibre content was associated with tryptophan metabolism, SNARE interactions and propanoate metabolism (Figure S2).

The study focused on the characterization of the genes of the pink module highly correlated to fibre, black module strongly linked to early, mid-season sugar content and grey linked to early-season sugar content. In this research, only the genes from modules positively correlated to these traits (black to early, mid-sugar content and pink to fibre) and with individual significative positive correlation to these traits were selected. The black module clustered 934 co-expressed genes, with 563 genes significantly positively correlated to early, mid-sugar content (CCS June) identified with 382 KEGG ID (Table S6) with sequences of the transcripts (Table S7). The pink module was represented with 924 co-expressed genes including 783 positively correlated to fibre content (Fibre September) identified with 301 (Table S8) with sequences of the transcripts (Table S9). On these 683 KEGG identified genes, 101 were present in the two modules (pink and black). The grey module of 15,929 genes was represented by 3,998 genes positively associated with early-season sugar content (CCS March).

**Fig. 7:**
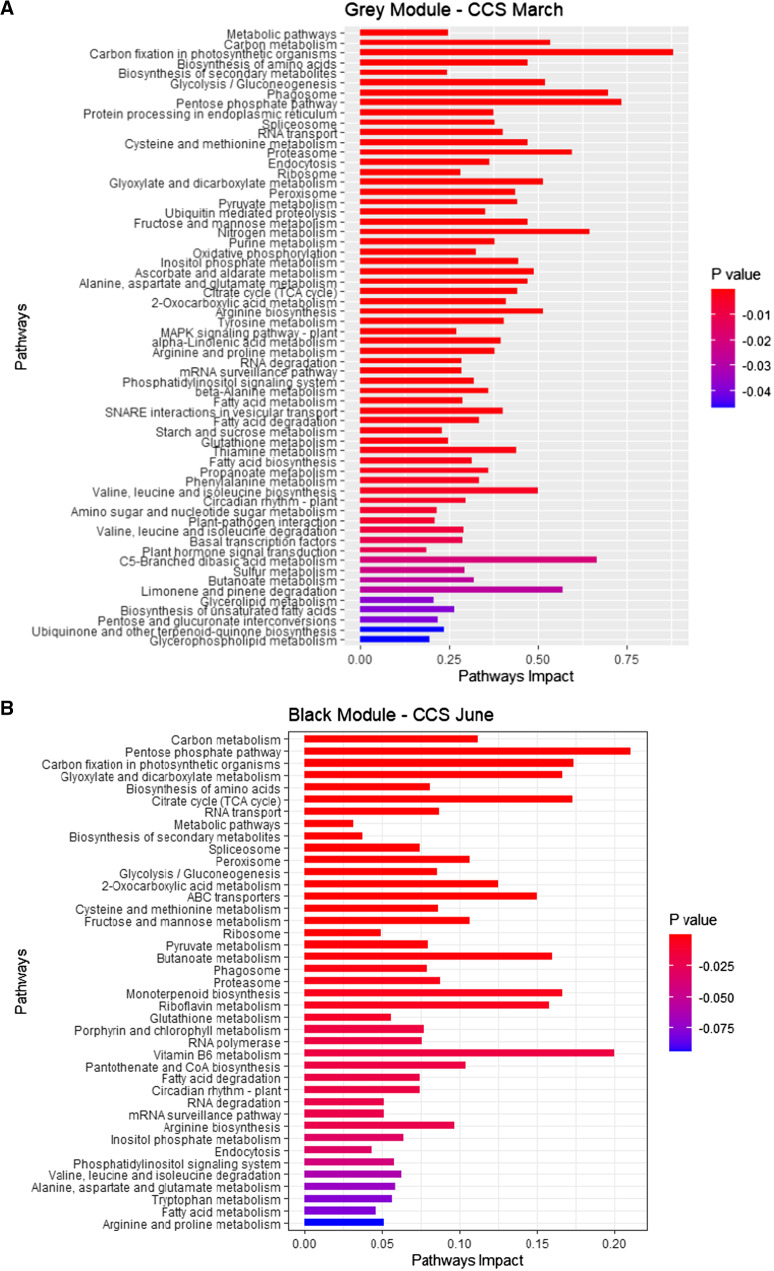

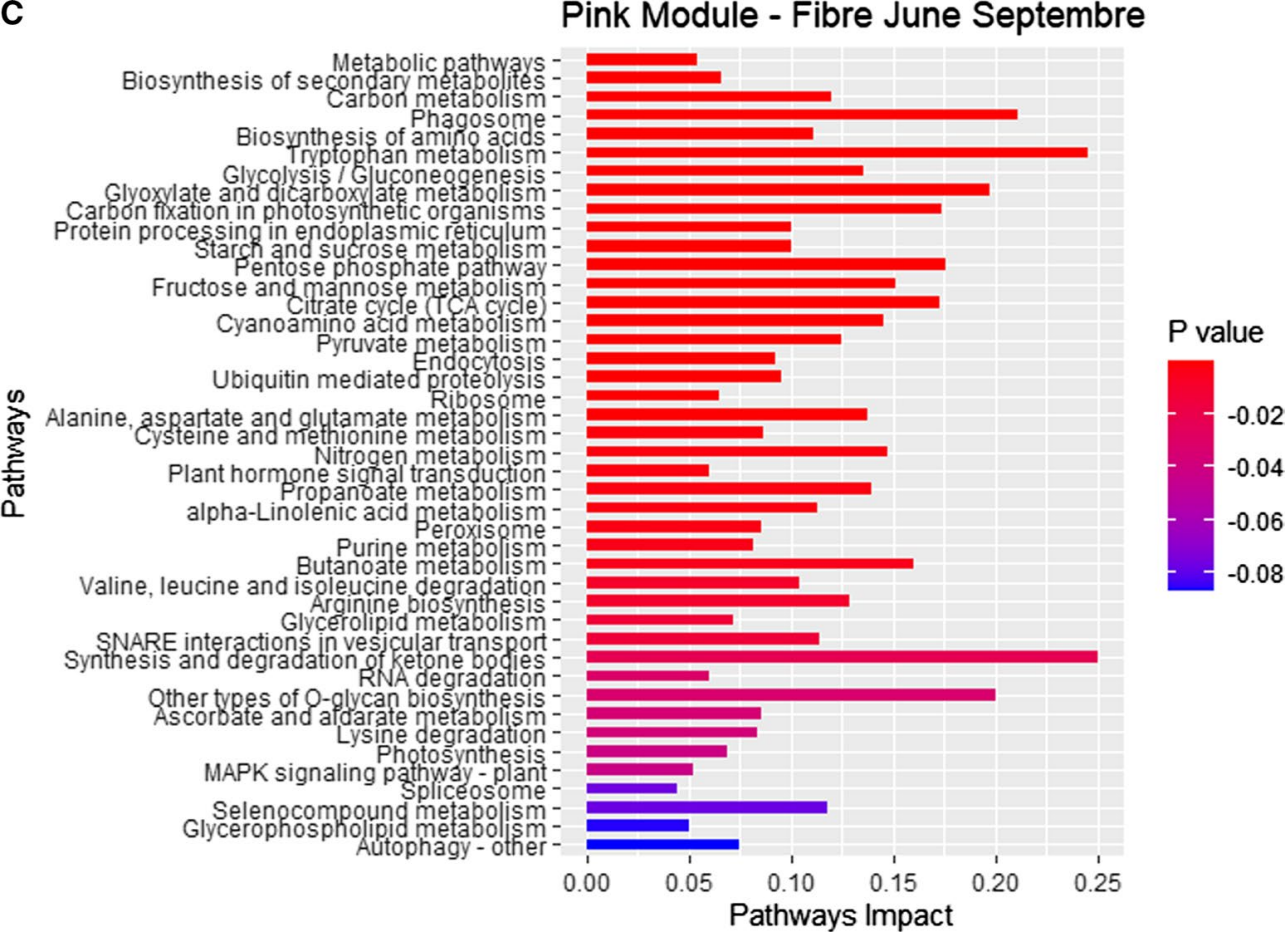
**A** KEGG pathways enrichment. Early-season sugar content (grey module), **B** KEGG pathways enrichment. Early-mid-season sugar content (black module), **C** KEGG pathways enrichment. Fibre content (pink module). “Pathways Impact” is the ration between the “Number of Genes” and the “Background” generated with KOBAS (Xie et al. 2011)

KEGG enrichment analysis showed the list of the metabolic pathways correlated to high early, mid-season sugar content and high fibre content. This KEGG pathway enrichment analysis revealed a positive correlation between early-season sugar content and the metabolic pathways carbon fixation in photosynthetic organisms, pentose phosphate pathway, phagosome and nitrogen metabolism (Fig. 7A). Comparison of enrichment pathways between co-expressed genes positively correlated to early, mid-season sugar content and co-expressed genes positively correlated to fibre content revealed common metabolic pathways such as carbon fixation in photosynthetic organisms, citrate cycle (TCA cycle), butanoate metabolism and cysteine and methionine metabolism. This KEGG pathway enrichment analysis also revealed strong different enriched pathways. Enrichment revealed that co-expressed genes linked to early, mid-season sugar content (black module) were more associated with the pentose phosphate pathway, vitamin B6 metabolism, citrate cycle (TCA) cycle and carbon fixation in photosynthetic organisms (Fig. 7B).

Co-expressed genes linked to fibre (pink module) were more linked to tryptophan metabolism, synthesis and degradation of ketone bodies, other types of O-glycan biosynthesis, phagosome and glyoxylate and dicarboxylate metabolism (Fig. 7C).

A focus on glycolysis / gluconeogenesis metabolism revealed a difference of attribution of the genes in this metabolism. The enzyme 6-phosphofructokinase was attributed to fibre content, while fructose-1,6-bisphosphatase I was linked to early, mid-season sugar content (Figure S3).

### COG functional annotation

The clusters of orthologous groups (COGs) of proteins were generated by comparing the protein sequences of complete genomes. COG analysis was processed using the black and pink modules. The black one composed of key co-abundant genes linked to high early-mid-season sugar content and the pink set of co-abundant genes linked to fibre content. Respectively, 11 and 18% of the genes linked to fibre and early-season sucrose had no function ascribed to them (Figure S4). Fifty percentage of the genes that are linked to these traits fell into six categories: post-translational modification, protein turnover, chaperones, transcription, signal transduction mechanisms, Translation, ribosomal structure and biogenesis, carbohydrate transport and metabolism.

The categories transcription, RNA processing and post-translational modification, protein turnover and chaperones were highly assigned to the trait early, mid-season sugar content. Subcategories linked to fibre content were mostly signal transduction mechanisms and carbohydrate transport and metabolism (Figure S4).

### Genes specifically correlated to high sugar content and to high fibre content

The most significant co-expressed genes correlated with early, mid-season sugar content (black module) included 6-phosphogluconate dehydrogenase, 1-phosphatidylinositol-3-phosphate 5-kinase, mannose-1-phosphate guanylyl transferase and arginase (Table S3).

Thirteen key genes with the most significance to early sugar content from the grey module were identified (Table 1 and Table S10). Genes from the most significant pathways were selected. Essential genes with the highest correlation with early-season sugar content were found in multiple essential pathways. The most significant genes in this group included fructose-bisphosphate aldolase, alcohol dehydrogenase and diacylglycerol diphosphate phosphatase / phosphatidate phosphatase (Table S10).

**Table 1:** Key genes positively correlated with early-season sugar

KEGG ID	Description [KEGG Enzyme ID]
K01623	Fructose-bisphosphate aldolase, class I [EC:4.1.2.13]
K00002	Alcohol dehydrogenase (NADP +) [EC:1.1.1.2]
K18693	Diacylglycerol diphosphate phosphatase / phosphatidate phosphatase [EC:3.1.3.81 3.1.3.4]
K01114	Phospholipase C [EC:3.1.4.3]
K03921	Acyl-[acyl-carrier-protein] desaturase [EC:1.14.19.2 1.14.19.11 1.14.19.26]
K00826	Branched-chain amino acid aminotransferase [EC:2.6.1.42]
K07897	Ras-related protein Rab-7A
K02940	Large subunit ribosomal protein L9e
K02868	Large subunit ribosomal protein L11e
K14486	Auxin response factor
K01100	Sedoheptulose-bisphosphatase [EC:3.1.3.37]
K02150	V-type H + -transporting ATPase subunit E
K01805	Xylose isomerase [EC:5.3.1.5]

Co-expressed genes specifically associated with fibre content included thioredoxin reductase (NADPH), uracil phosphoribosyltransferase, triose/dihydroxyacetone kinase / FAD-AMP lyase (cyclizing), (DAK) and phosphoglycerate kinase and phosphoglycerate kinase (PGK) (Table S4).

## Discussion

The expression profiles from internodes at different stages of development differed significantly. The genes that change most significantly were associated with cell expansion, cell wall and lignin biosynthesis. The differences in gene expression between the genotypes account for a larger portion of the total variance. This finding was particularly interesting and surprising, taken the very narrow genetic base of sugarcane and the very high selective pressure on CCS.

This study revealed large, interconnected pathways related to high fibre and high sugar content. Two significant groups of co-expressed genes were identified and genes functionally annotated. One of them was positively correlated to early, mid-season sugar content and negatively correlated to fibre content. The other was negatively correlated to early, mid-season sugar content and positively correlated to fibre content. Analysis of these two groups displayed the genes positively correlated to fibre were assigned to the KEGG pathways such as tryptophan metabolism, synthesis and degradation of ketone bodies, other types of O-glycan biosynthesis and glyoxylate and dicarboxylate metabolism. Genes positively correlated to high early-season sugar content were found to be allocated to carbon fixation in photosynthetic organisms, pentose phosphate pathway and phagosome. Key genes highly specifically positively correlated to high early-season sugar content fibre content that may be used as biomarkers were fructose-bisphosphate aldolase, alcohol dehydrogenase, diacylglycerol diphosphate phosphatase, phospholipase C and acyl-desaturase. Key genes highly specifically positively correlated to fibre content such as thioredoxin reductase, uracil phosphoribosyltransferase, dihydroxyacetone kinase, phosphoglycerate kinase (K00927), adenylate kinase and beta-glucosidase were identified.

Results illustrated that genotypes and development stages are key factors controlling transcript expression. The dynamics of the transcriptome during development revealed that gene expression was different between internode maturity and seasonality. The position of the internode was the most significant, revealing the highest similarity between internodes with the same position (same maturity), such as internodes 5 at different ages than between internodes with similar age but different maturity (Fig. 1). These results highlighted an overall decrease in gene expression with age and maturity of the plant. As development progressed, down-regulated genes were continuously in higher proportion than up-regulated genes. This result indicated a reduction of cellular and metabolism activity with age in sugarcane.

Interestingly, unsupervised hierarchical clustering of gene expression revealed a stronger level of segregation associated with the genotypes than developmental stage. The 24 genotypes were very clearly segregated showing the high performance of the transcriptome analysis and confirming the quality of the data. DEG analysis using Illumina RNA-Seq is a powerful approach for processing data in polyploid species. The combination of PCA and hierarchical clustering led to the capture of different levels of differentiation.

Phosphofructokinase (PFK) is a key enzyme of glycolysis, the metabolic pathway that converts glucose to pyruvate. PFK is involved in the conversion of fructose 6-phosphate and adenosine triphosphate (ATP) to fructose 1,6-bisphosphate I and adenosine diphosphate (ADP) (Hubert 1986; Givan 1999; Plaxton and Podestá, 2007). Reversely the degradation of fructose 1,6-bisphosphate I involves the fructose-1,6-bisphosphatase I (FBPase), the essential enzyme of the gluconeogenesis. This reaction converts fructose-1,6-bisphosphate and H2O to fructose 6-phosphate and Pi. The results of this study show a specific positive correlation between fructose 1,6-bisphosphate I and high early, mid-season sugar content. These results are consistent with the gluconeogenesis function of this enzyme. Our results also suggest that PFK activity is more influenced by genotype than by development. In agreement with our results, the PFP/PFK ratio was found to be genetically determined in Daurus carota cell lines, (Krook et al. 2000). Studies on sucrose storage described did not show a relationship between PFK activity and sucrose content (Whittaker and Botha 1999). In our research, PFK was not linked to sugar content but was linked to fibre content. PFP was reported to vary significantly between sugarcane varieties and inversely correlated with the sucrose content (Whittaker and Botha 1999). In this study, PFP was positively correlated with sucrose content and also with fibre content. Studies on transgenic plants, with PFP down-regulation showed no significant differences in growth and development with a significant increase of sucrose in the immature internodes but not in the mature internodes (Groenewald et Botha 2008).

Collectively, the Tucker3 results show that at least 86.9% of the variation in sugarcane gene expression profiles could be attributed to internode and genotype main effects, with no evidence of substantial interaction between the two factors. This has important implications both for basic understanding of this biological system and breeding purposes.

The selection of varieties with specific sugar maturity profiles is essential for harvest management to maximize CCS maturity and TCH. The goal of the project was to characterize candidate genes and metabolic pathways associated with high early sugar content varieties and fibre content. The transcriptome analysis described pools of genes directly linked to the different traits, such as early, mid-season sugar content, fibre content and TCH. This analysis revealed a strong similarity of gene expression between early-season sugar content cultivars KQ228, Q253, Q240 and SRA8. WGCNA analysis showed that high early-season sugar content was related to two modules (black and grey). The first grouped co-expressed genes principally from pentose phosphate pathways, vitamin B6 metabolism, carbon fixation in C4-dicarboxylic acid cycle, TCA Cycle and glyoxylate and dicarboxylate metabolism. The second group included genes mostly in carbon fixation in C4-dicarboxylic acid cycle, pentose phosphate pathway, phagosome, nitrogen. A high positive correlation between early-season sugar content cultivars and the pentose phosphate pathway (PPP) was demonstrated. The results are consistent with the fact that the PPP is a major part of glucose metabolism where fructose 6-phosphate (F6P) and glyceraldehyde 3-phosphate (G3P) are generated using glucose 6-phosphate (G6P) (Gumaa and McLean 1969; Ramos-Martinez 2017; Ge et al. 2020).

Citrate cycle, also known as the Krebs cycle and tricarboxylic acid (TCA) cycle, has a major role in respiratory metabolism of photosynthetic and heterotrophic plant organs (AraÚJo et al. 2012; Janssen et al. 2019). Several B vitamins are involved as cofactors in the TCA cycle. Glyoxylate and dicarboxylate metabolism was also found to be positively correlated with early-season sugar content. The pathways involve succinic acid and α-oxoglutaric acid, two essential components of the TCA cycle that connect glyoxylate and dicarboxylate metabolism to six other pathways (Liu et al. 2016).

COG analysis revealed a strong correlation between the categories transcriptional, RNA processing and modification and post-translational modification, protein turnover, chaperones and high early-season sugar content. All these results may indicate an intense early transcriptional and carbon fixation activity in the varieties with early-season sugar content.

Eight enzymes were identified to play the most influential regulatory roles associated with high early-season sugar content. Four of them, fructose-bisphosphate aldolase, alcohol dehydrogenase (NADP +), sedoheptulose-bisphosphatase and xylose isomerase are important enzymes involved in multiple metabolisms such as carbon fixation in photosynthetic, pentose phosphate pathway, fructose and mannose metabolism and carbon metabolism. These results support previous studies on decreased photosynthetic capacity and alteration of carbohydrate accumulation linked to a reduction of sedoheptulose-1,7-bisphosphatase (Harrison et al. 1998; Tamoi et al. 2001; Mitchell et al. 2020). Four others—diacylglycerol diphosphate phosphatase/phosphatidate phosphatase and phospholipase C are involved in glycerophospholipid metabolism, acyl-desaturase in fatty acid biosynthesis and branched-chain amino acid aminotransferase in the biosynthesis of amino acids. These genes may be selected as potential targets for metabolism engineering and high early-season sugar content biomarkers. Several phospholipase C have been described as potential candidates for genetically engineering for stress tolerance and crop productivity (Singh et al. 2015). The variety KQ228 is a prime example of a variety requiring further investigation to validate these results and elucidate the complex biological function of these genes in carbon partitioning.

Glyceralhehyde-3P to D-fructose 6P was linked to enzymes related to early, mid-sugar content and to the conversion of glyceralhehyde-3P to glycerone-P, step before the gluconeogenesis by the enzymes linked to high fibre content. The enzymes, ß-glucosidase, phenylalanine/tyrosine ammonia-lyase (PTAL) and phenylalanine ammonia-lyase (PAL) are involved in the first steps of phenylpropanoid biosynthesis. PAL have been shown to be linked to lignin content in arabidopsis and sugarcane (Kasirajan et al. 2018; Xi et al. 2018). PTAL has been described to be linked to grass cell walls (Barros et al. 2016). Beta-glucosidase is known to release glucose by hydrolysis of beta-D-glucosides and oligosaccharides (Morant et al. 2008). Caffeic acid 3-O-methyltransferase (COMP) has been described to be linked to lignin (Ma et al. 2008). In this study, COMP is highly linked to fibre content and more surprisingly to early, mid-season sugar content.

This study revealed a complex, interconnected and dynamic biological system linked to sugar and fibre content that has not been reported before. Modules of genes positively correlated to early- or mid-season sugar content were negatively correlated to fibre content and reciprocally modules of genes positively correlated to fibre content were negatively correlated to sugar content. This characteristic may explain the difficulty of breeding high early-season sugar content and high fibre content. Many studies have been realized to characterize the genes and reveal the lignin biosynthesis in sugarcane (Bottcher et al. 2013; Guzzo de Carli Poelking et al. 2015; Vicentini et al. 2015; Ferreira et al. 2016; Hoang et al. 2017a; Kasirajan et al. 2018; Jardim-Messeder et al. 2020) and sucrose accumulation (Whittaker and Botha 1997; Chen et al. 2019). However, knowledge of gene expression in interconnected metabolism pathways linked to fibre and sugar content requires further investigation.

Key genes, key metabolic pathways and their interconnections leading to early-season sugar content and yield was possible by the generation for this study an extensive sequencing and a large phenotypic dataset. Bioinformatics tools such as WGCNA provide complementary information to traditional DEG analysis, revealing the complexity of co-expressed and correlated genes which can be involved in the same pathways (Michalak 2008; Serin et al. 2016; Hoang et al. 2017b; Thirugnanasambandam et al. 2017). Omics data integration and analysis of complex networks of biological function reveal links of specific genetic, physical, physiological and chemical properties (Hawe et al. 2019). Similar genes are involved in a wide range of metabolic function as found with other studies (Xu et al. 2018; Fang et al. 2019).

This study using correlation networks proved to be a powerful way to investigate the regulation of carbon allocation, partitioning and metabolism regulation linked to sugar and fibre content in a polyploid plant. This research described and compared in detail the links between traits, which may be a guide for breeders and growers to use in choosing the varieties that will deliver their requirements. The results allow a hypothesis to be developed on the identity of genes that could provide a blueprint for selecting desirable high sugar varieties.

## Supplementary Information

Below is the link to the electronic supplementary material.

Supplementary file1 (XLSX 864 KB)

Supplementary file2 (DOCX 483 KB)

## Declarations

### Data availability

The data for this study have been deposited in the European Nucleotide Archive (ENA) at EMBL-EBI under accession number PRJEB44480 (https://www.ebi.ac.uk/ena/browser/view/PRJEB44480).
