# Supplementary material for: Transcriptome changes in the developing sugarcane culm associated with high yield and early-season high sugar content
Source: Theor Appl Genet. 2022 Feb 27;135(5):1619–36. doi: 10.1007/s00122-022-04058-3 (PMC9110458; doi:10.1007/s00122-022-04058-3)
Supplement: Supplementary file 2 — Supplementary file2 (DOCX 483 KB) [file 122_2022_4058_MOESM2_ESM.docx]

**Supporting figures**

**Figure S1** Workflow of WGCNA analysis and genes identification.

**Figure S2** KEGG Pathways enrichment for each of the modules of the trait relationship analysis. Hierarchical clusters of the colour modules, respectively ME green, ME yellow, ME turquoise, ME red, ME black, ME magenta, ME pink, ME grey, ME blue and ME brown were represented on the top of the heatmap. Hierarchical clusters of the metabolic pathways were displayed on the left of the heatmap. Ward clustering algorithm with Euclidean distance measure was used with not normalised data to take into consideration the level of expression.

**Figure S3** Detail of the Glycolysis / Gluconeogenesis metabolism involving enzymes linked to fibre content (6-phosphofructokinase 1 [EC:2.7.1.11]) and early, mid-season sugar content (fructose-1,6-bisphosphatase I [EC:3.1.3.11]).

**Figure S4** COG functional classification of the genes associated to Fibre September (pink colour) and to early, mid-season sugar content (black colour).


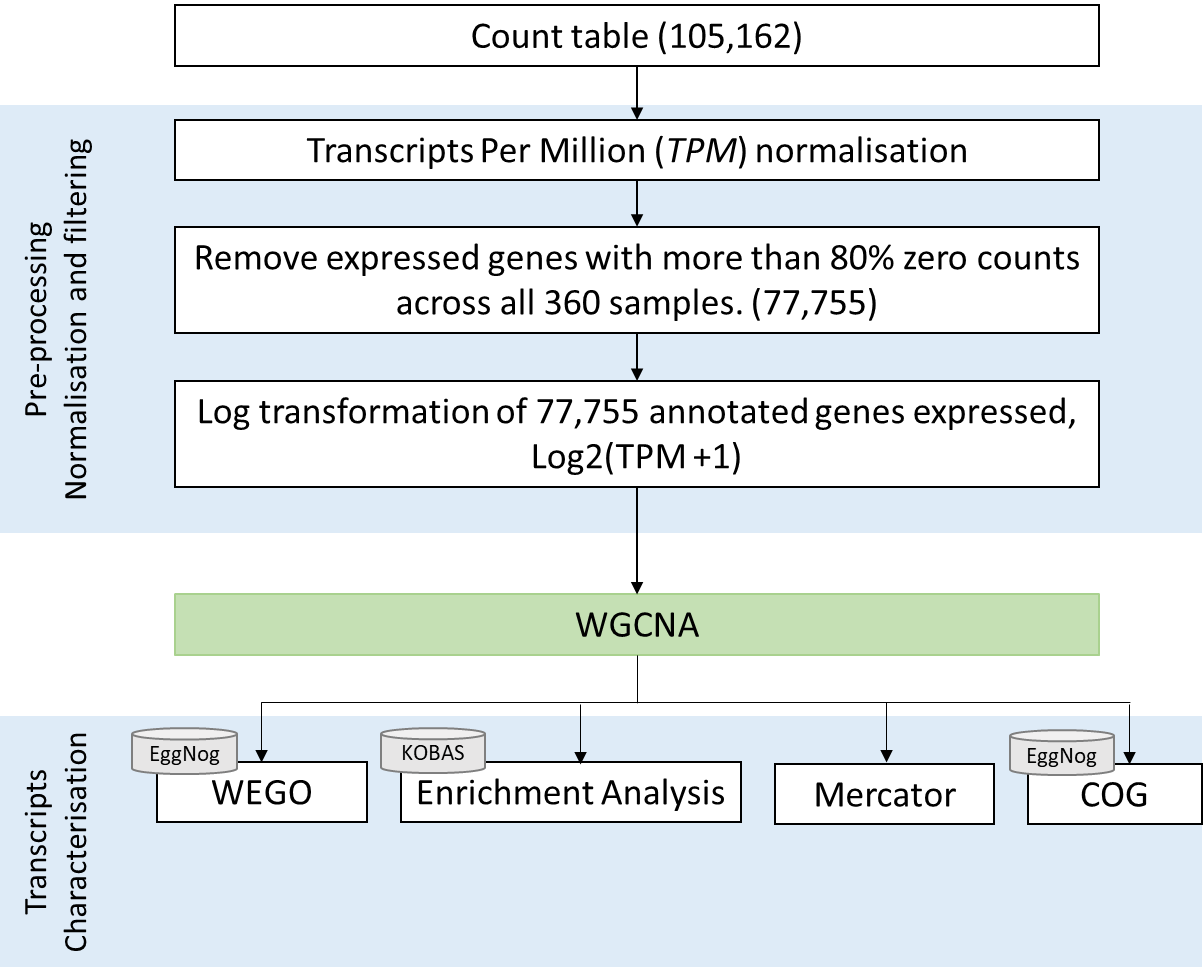


**Figure S1**


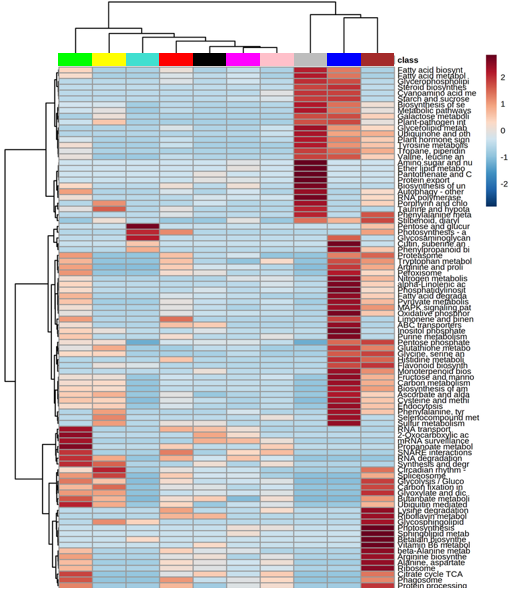


**Figure S2**


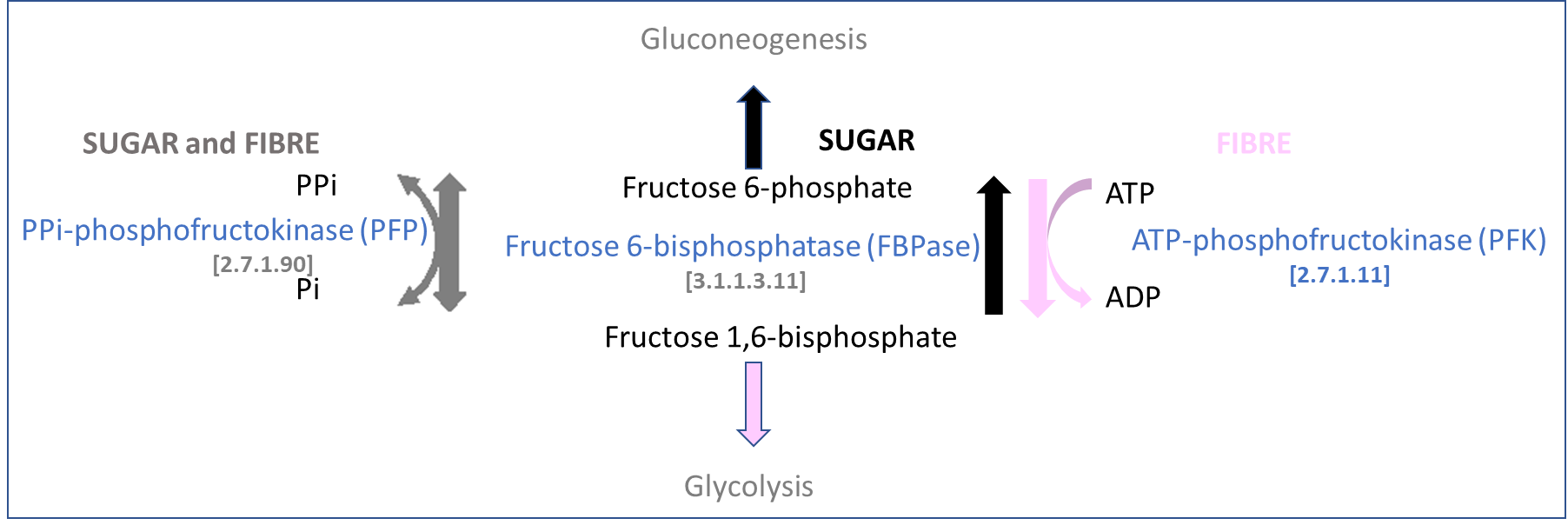


**Figure S3**


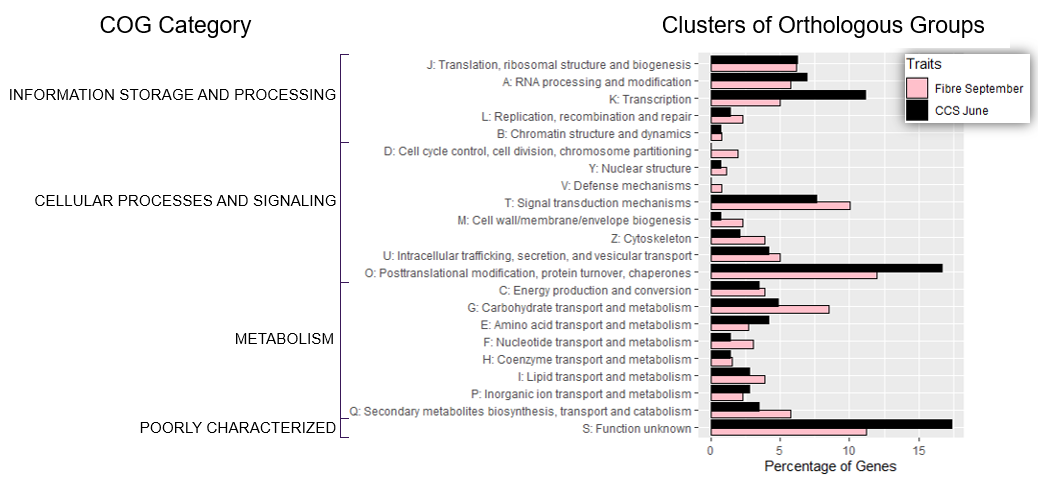


**Figure S4**
